# Cytotoxicity and Apoptotic Mechanism of 2-Hydroxyethyl Methacrylate via Genotoxicity and the Mitochondrial-Dependent Intrinsic Caspase Pathway and Intracellular Reactive Oxygen Species Accumulation in Macrophages

**DOI:** 10.3390/polym14163378

**Published:** 2022-08-18

**Authors:** Chien-Ying Lee, Yung-Chuan Ho, Shiuan-Shinn Lee, Yi-Ching Li, Mei-Yu Lai, Yu-Hsiang Kuan

**Affiliations:** 1 Department of Pharmacology, School of Medicine, Chung Shan Medical University, Taichung 40201, Taiwan; 2 Department of Pharmacy, Chung Shan Medical University Hospital, Taichung 40201, Taiwan; 3 School of Medical Applied Chemistry, Chung Shan Medical University, Taichung 40201, Taiwan; 4 School of Public Health, Chung Shan Medical University, Taichung 40201, Taiwan; 5 Department of Nursing, Chung Shan Medical University Hospital, Taichung 40201, Taiwan; 6 School of Nursing, Chung Shan Medical University, Taichung 40201, Taiwan

**Keywords:** HEMA, RAW264.7 macrophages, cytotoxicity, genotoxicity, apoptosis, caspases

## Abstract

Macrophages are mainly active cells of the immune system and play a role in the defense of pathogens. However, the overactivation of macrophages by fatal pathogens can result in toxic responses. 2-hydroxyethyl methacrylate (HEMA), which is a hydrophilic monomer, is used in dental adhesive reagents and composite resins as well as biocompatible hydrogels. The mechanisms underlying the genotoxicity engendered by HEMA-induced apoptosis that leads to cytotoxicity remain unclear. Accordingly, this study was conducted to clarify such mechanisms. The results showed that HEMA induced cell toxicity in RAW264.7 macrophages depending on the concentration. A higher HEMA concentration was associated with a higher level of apoptosis and genotoxicity. Moreover, HEMA induced a concentration-dependent increase in mitochondrial dysfunction and the intrinsic caspase pathway, including the activation of caspase-3 and caspase-9. HEMA was also found to upregulate intracellular reactive oxygen species generation and to decrease the activity of antioxidant enzymes, including superoxide dismutase and catalase. Taken together, the mitochondrial-dependent intrinsic caspase pathway and intracellular reactive oxygen species accumulation were found to mediate HEMA-induced genotoxicity and apoptosis, leading to cytotoxicity in RAW264.7 macrophages.

## 1. Introduction

Macrophages are the active cells of the innate immune system. They phagocytose bacteria and secrete pro-inflammatory mediators, thus contributing to the defense against pathogens [1]. Macrophages are present in all vertebrate tissues and are involved in hemostasis. Resident macrophages in the liver, brain, lung, and epidermis are called Kupffer cells, microglia, alveolar macrophages, and Langerhans cells, respectively [2]. When faced with dangerous pathogens that appear foreign, macrophages are activated to impart toxic effects to self- and peripheral tissues [3,4]. The formation of atherosclerotic plaque, viral infection, inflammation, and sepsis are associated with macrophage death [5]. Pathological pathways regulate macrophage cytotoxicity, including apoptosis and necrosis via genotoxicity [5,6,7]. The molecular mechanisms underlying these toxic effects involve caspase activation, mitochondrial dysfunction, the generation of reactive oxygen species (ROS), and the depletion of antioxidative enzymes (AOEs) in macrophages [5,6,7,8].

2-hydroxyethyl methacrylate (HEMA), a hydrophilic monomer, can be used to create poly(2-hydroxyethylmethacrylate), a polymer, as well as to produce biomaterials such as dental adhesive reagents, dental composite resins, and biocompatible hydrogels [9,10]. HEMA is released from the aforementioned biomaterials in the presence of differential solvents on the first day after polymerization [11]. Because dental biomaterials are highly permeable, the peripheral tissues and cells can be harmed by the leached HEMA [12]. HEMA induces cytotoxicity and apoptosis in macrophages, macrophage-like osteoclasts, and alveolar macrophages [13,14,15,16]. The overgeneration of ROS and the depletion of AOEs are the main factors affecting HEMA-induced apoptosis through caspase-3 activation in macrophages [16,17,18,19]. However, the mechanisms underlying the genotoxicity engendered by HEMA-induced apoptosis, which ultimately leads to cytotoxicity, remain unknown. Consequently, the objective of the present study was to evaluate whether the pathways that depend on mitochondrial disruption and the accumulation of ROS within the RAW264.7 macrophages are involved in the cytotoxicity and genotoxicity caused by apoptosis after HEMA treatment.

## 2. Materials and Methods

### 2.1. Materials

Fetal bovine serum (FBS), antibiotic–antimycotic solution, Dulbecco’s modified Eagle’s medium (DMEM), and phosphate-buffered saline (PBS) were obtained from Gibco BRL, Life Technologies (Grand Island, NY, USA). The Annexin V-FITC apoptosis-detection kit, JC-1, HEMA, dichlorofluorescin diacetate (DCFH-DA) and other chemical reagents, unless specifically stated, were purchased from Sigma-Aldrich (St. Louis, MO, USA). The caspase-3 fluorometric assay kit, caspase-8 fluorometric assay kit, and caspase-9 fluorometric assay kit were obtained from Enzo Life Sciences (Farmingdale, NY, USA). The catalase (CAT) assay kit and superoxide dismutase (SOD) assay kit were obtained from Cayman Chemical (Ann Arbor, MI, USA). The HEMA solution was derived from dimethyl sulfoxide (DMSO), with the final concentration being less than 0.5% (v/v) nontoxic.

### 2.2. Cell Culture and Treatment

RAW264.7 cells (BCRC No.6001) are murine macrophage cells derived from BALB/c mice extracted from the Bioresources Collection and Research Center in Hsinchu, Taiwan. The RAW264.7 cells were cultured in DMEM supplemented with 10% FBS, 1 mM sodium pyruvate, and 1% antibiotic–antimycotic solution—which contains penicillin, streptomycin, and Amphotericin B—and incubated at 37 °C in a humidified atmosphere of 5% CO_2_ [20]. After seeding for the night, the cells were incubated in HEMA for 24 h at concentrations of 0, 0.5, 1, 5, and 10 mM. The cells were then harvested and used for further experiments.

### 2.3. Cell Viability Assay

The effect of HEMA on the viability of the RAW 264.7 cells was determined using the 3-(4,5-dimethylthiazol-2-yl)-2,5-diphenyl tetrazolium bromide (MTT) assay, as described in our previous study [20]. In brief, the 1 × 10^6^ cells were incubated with various concentrations of HEMA (0, 0.5, 1, 5, and 10 mM) for 24 h, then 0.5 mg/mL of MTT were added to each well. After incubation for 4 h, the reaction was completed by adding the DMSO solution. The absorption was detected with a 570 nm microplate reader (Synergy HT, BioTek, Winooski, VT, USA). The percentage of cell survival was calculated by using the following formula: (absorbance of cells treated with HEMA − absorbance of blank well/absorbance of cells treated without HEMA − absorbance of blank well) × 100%.

### 2.4. Flow Cytometric Analysis of Apoptosis and Necrosis

The effects of HEMA on apoptosis and necrosis were determined using the Annexin V-FITC apoptosis-detection kit, as described previously [20]. After 24 h of incubation with different HEMA concentrations, the cells were collected via trypsinization and washed with PBS. The 1 × 10^6^ cells were dyed for 30 min at room temperature with the 100 μL binding buffer that contained the 5 μL of Annexin V–FITC and 5 μL of PI. The acquisition of the RAW264.7 cells and the data analysis were measured by the Accuri C6 flow cytometer (BD Biosciences, San Jose, CA, USA). The percentages of cells in the viable, necrotic, and apoptotic cells were presented as annexin-V-FITC-negative and PI-negative, PI-positive, and annexin-V-FITC-positive, respectively. 

### 2.5. Micronucleus Assay

Micronucleus (MN) formation, as the marker of DNA damage, was measured by the alkaline cytokinesis–block MN assay, as described above [21]. In short, the 1 × 10^6^ cells were incubated with different concentrations of HEMA and 3 mg/mL of cytochalasin B for 24 h. After incubation, the cells were washed, incubated with 75 mM KCl, fixed in the methanol/acetic acid mixture, and stained with the 3% Giemsa solution. The MN were observed using light microscopy.

### 2.6. Single-Cell Gel Electrophoresis

DNA damage was analyzed using the alkaline single-cell gel electrophoresis (Comet) assay, as described above [21]. After treatment, 1 × 10^6^ cells were mixed with the solutions of low-melting-point agarose and placed in a microscopic slide that was pre-loaded with normal-melting-point agarose after treatment. The slides were incubated with the alkaline lysis buffer, which contained 2.5 M NaCl, 100 mM EDTA, 10 mM Tris pH 10, 1% Triton X-100, 200 mM NaOH, 34.1 mM N-Lauroyl-Sarcosine, and 10% DMSO, at 4 °C for 1 h. The slides were then washed, electrophoresed, neutralized, stained with ethidium bromide, and finally analyzed using the image-analysis software Comet v. 3 (Kinetic Imaging Ltd., Liverpool, UK).

### 2.7. Flow Cytometric Analysis of Mitochondrial Dysfunction

Mitochondrial dysfunction induced by HEMA was evaluated using JC-1 staining, as described previously [21]. In brief, after incubation with HEMA, the cells were collected and stained with 10 mg/mL JC-1 at 37 °C for 30 min. The cells were washed with PBS, and the collected cells and data were analyzed using the BD Accuri C6 flow cytometer with C6 software.

### 2.8. Caspase Activation Assay

The activation of caspase-3, -8, and -9 induced by HEMA was determined by the caspase fluorometric assay kits, as described above [21]. The lysis buffer and reaction buffer were obtained from caspase fluorometric assay kits. In short, the cells were collected and lysed with lysis buffer after treatment with HEMA. An equal amount of protein was extracted from each sample and incubated with the fluorogenic substrates including DEVD-AFC, IETD-AFC, and LEHD-AFC for caspase-3, caspase-8, and caspase-9 in reaction buffer, respectively. After incubation at 37 °C for 2 h and upon excitation at 485 nm, the fluorescence intensity was measured at 505 nm using a fluorescence microplate reader (BioTek Instruments, Winooski, VT, USA).

### 2.9. AOE Activition Assay

The effect of HEMA on the activation of AOEs, including CAT and SOD, was evaluated by CAT and SOD assay kits in accordance with the manufacturer’s protocol and previous studies, respectively [20].

### 2.10. Intracellular ROS-Generation Measurement

The intracellular levels of ROS induced by HEMA were evaluated using the DCFH-DA assay, as described above [20]. In brief, the cells were incubated with 5 μM DCFH-DA for 0.5 h at 37 °C in the dark after treatment with HEMA. Fluorescence was determined at 485/530 nm using a fluorescence microplate reader (BioTek Instruments).

### 2.11. Statistical Analysis

All experiments were carried out at least three times. The data are represented as an average of the standard deviation (SD). Statistical comparisons were made using the one-way analysis of variance followed by the Bonferroni post-hoc test. Data were analyzed using SPSS software (IBM, Armonk, NY, USA). The *p*-value was considered statistically significant if less than 0.05.

## 3. Results

### 3.1. HEMA’s Effects on the Cellular Viability of RAW264.7 Cells

**Figure 1:**
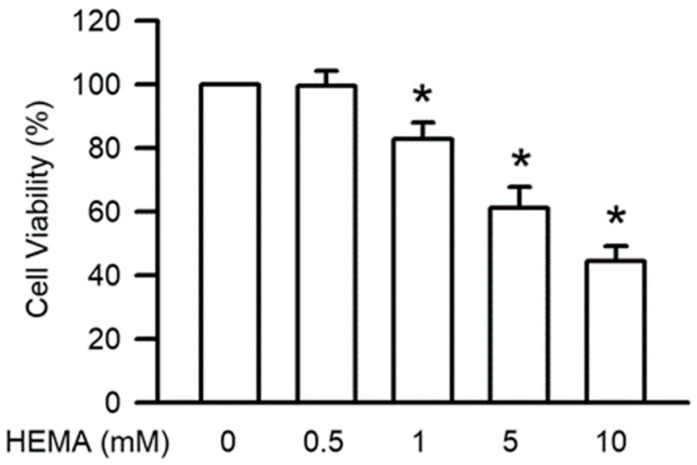
HEMA reduced cell viability in RAW264.7 cells. Cell survival was measured by the MTT test after 24 h of HEMA incubation. The value is expressed as the percentage of control cells treated with the vehicle. Results are expressed as means ± SD. The number of parallel measurements is 5. * means that *p* < 0.05 compared with HEMA at the concentration of 0 mM.

To assess the cell survival of HEMA in RAW264.7 macrophage cells, the cells were treated for 24 h with HEMA at different concentrations. The results of the MTT assay (Figure 1) revealed that HEMA significantly reduced the function of RAW264.7 cells, depending on the concentration. Significant decreases in cell viability were observed at the concentration of 1 mM (*p* < 0.05).

### 3.2. HEMA’s Effects on Necrosis or Apoptosis of RAW264.7 Cells

**Figure 2:**
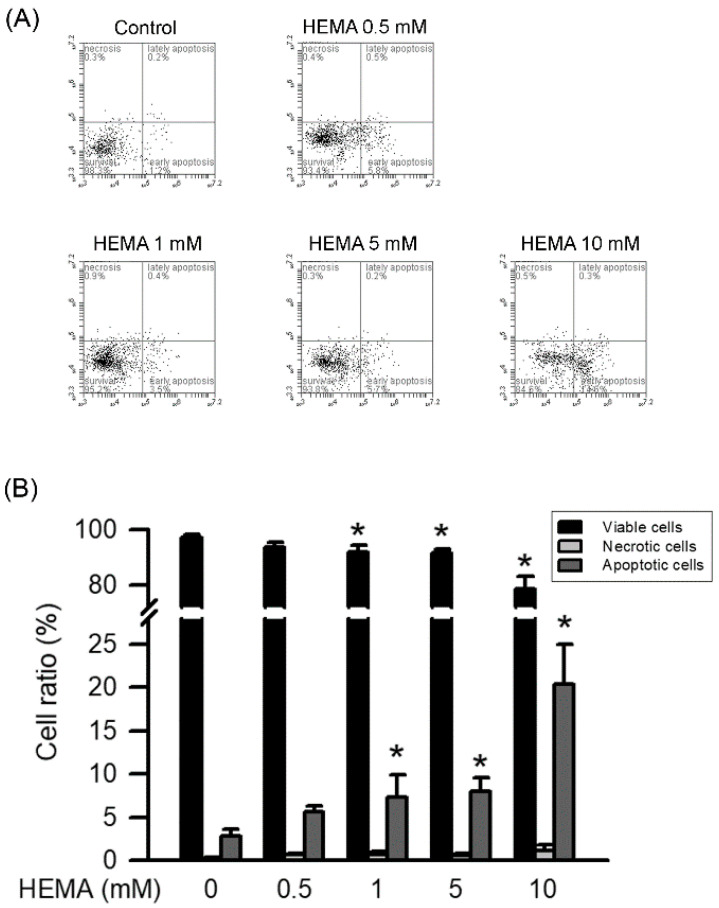
HEMA induced apoptosis in RAW264.7 cells. After treatment, RAW264.7 cells were collected, stained with Annexin V-FITC and PI, and then analyzed by dual-staining flow cytometry. (**A**) Representative FACS plot of RAW264.7 macrophages incubated with HEMA. (**B**) Quantitatively, the percentage of viable cells (black column), necrotic cells (gray column), and apoptotic cells (dark gray column) was calculated and analyzed. Data are expressed as mean ± SD. The number of parallel measurements is 4. * means that *p* < 0.05 compared with HEMA at the concentration of 0 mM.

The RAW264.7 cells labeled with annexin V-FITC and PI were used to identify the necrosis or apoptosis induced by HEMA. As illustrated in Figure 2 and Table 1, HEMA induced apoptosis in the RAW264.7 cells in a concentration-dependent manner. Significant increases in apoptosis were observed at the concentration of 1 mM (*p* < 0.05). On the other hand, necrosis was not caused by HEMA at the concentration of 10 mM in the RAW264.7 macrophages.

### 3.3. HEMA’s Effects on Genotoxicity in RAW264.7 Macrophages

**Figure 3:**
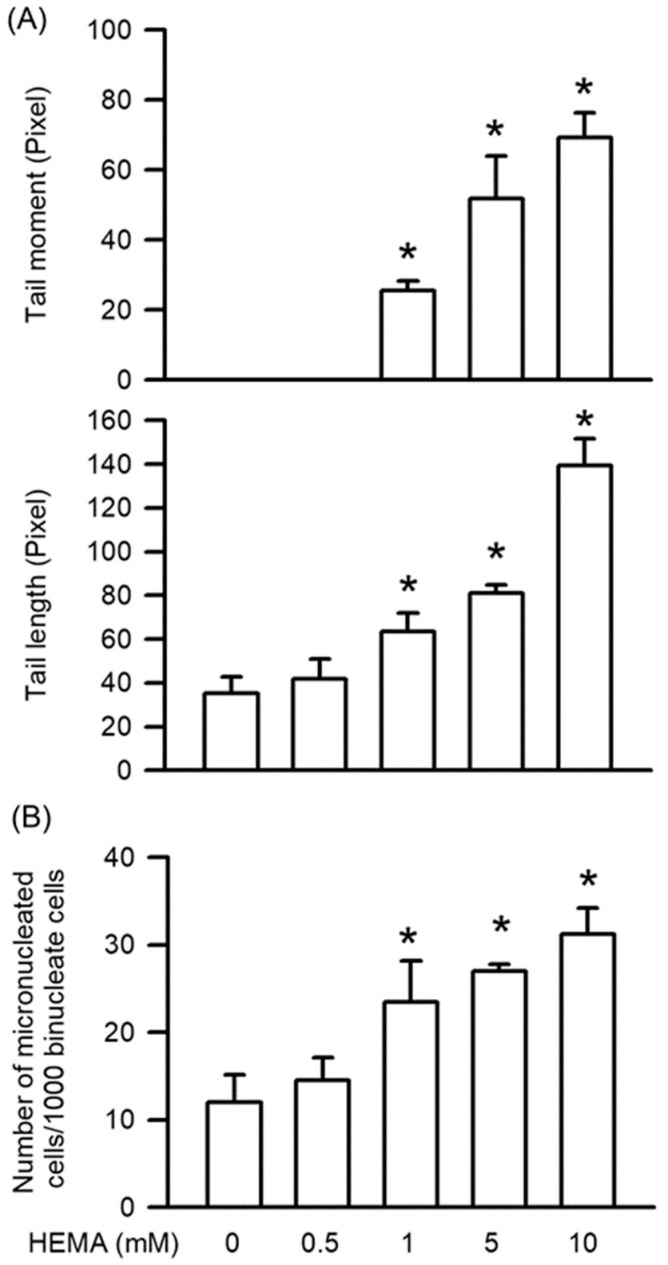
HEMA induced genotoxicity in RAW264.7 cells. (**A**) After the Comet assay, genotoxicity was quantified as tail moment and tail length. (**B**) After the MN assay, genotoxicity was quantified as MN formation. Data are expressed as mean ± SD. The number of parallel measurements is 4. * means that *p* < 0.05 compared with HEMA at the concentration of 0 mM.

Genotoxicity, which also means DNA damage, is the major inducer of apoptosis. As shown in Figure 3, HEMA-induced genotoxicity was measured by the Comet and MN assays. The results of the Comet assay indicated that compared to those of the control cells, the tail moment and tail length values in the HEMA-treated cells were significantly increased in a concentration-dependent manner (starting from 1 mM; *p* < 0.05; Figure 3A). According to the MN assay results, MN formation was significantly increased in the HEMA-treated cells compared with that in the control cells in a concentration-dependent manner (starting from 1 mM; *p* < 0.05; Figure 3B).

### 3.4. HEMA’s Effects on the Activation of Caspase-3, Caspase-8, and Caspase-9 in RAW264.7 Macrophages

**Figure 4:**
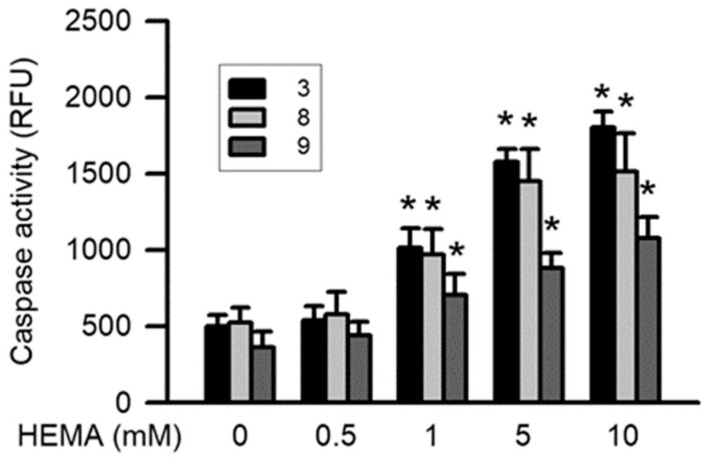
HEMA induced activation of caspase-3, caspase-8, and caspase-9 in RAW264.7 macrophages. Activation of caspase-3 (black column), caspase-8 (gray column), and caspase-9 (dark gray column) was measured by the caspase fluorometric assay kits after 24 h of HEMA incubation. Data are expressed as means ± SD. The number of parallel measurements is 5. * means that *p* < 0.05 compared with HEMA at the concentration of 0 mM.

Caspase-3, caspase-8, and caspase-9 play an important role in apoptosis induced by genotoxicity. After treatment for 24 h, the HEMA-induced activation of caspase-3 and caspase-9 was significantly dependent on concentration (starting from 1 mM; *p* < 0.05; Figure 4). Moreover, significant HEMA-induced caspase-8 activity was observed (starting from 10 mM; *p* < 0.05; Figure 4).

### 3.5. Effects of HEMA on Mitochondrial Dysfunction in RAW264.7 Macrophages

**Figure 5:**
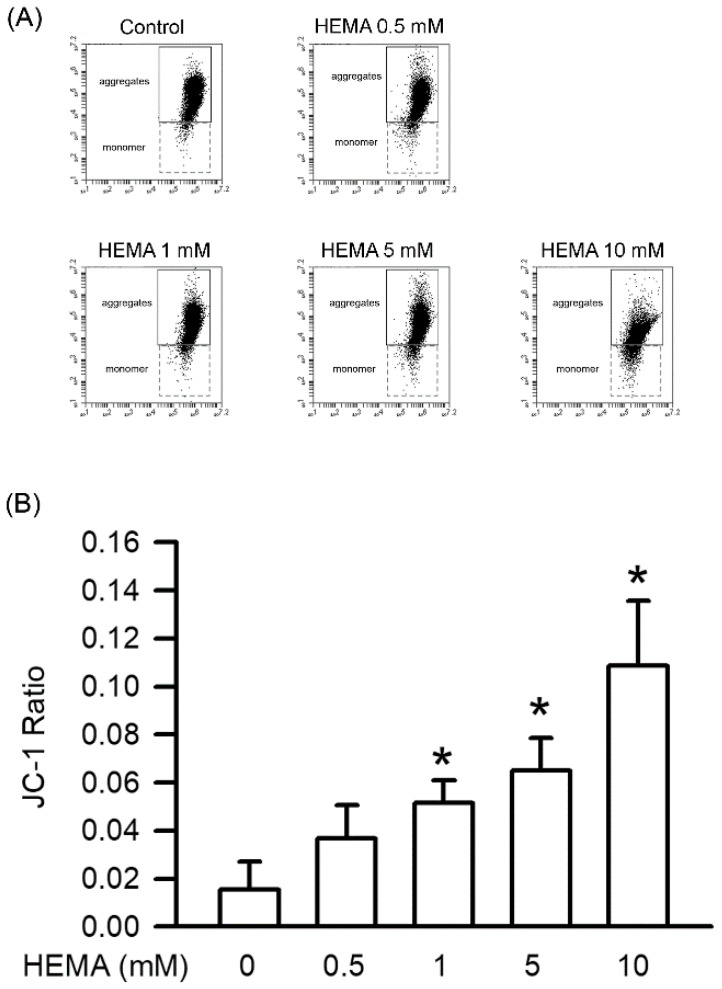
HEMA caused mitochondrial dysfunction. The mitochondrial dysfunction was measured with JC-1 after 24 h of HEMA treatment. (**A**) The original flow cytometry plot showing the evaluation of the mitochondrial dysfunction in RAW264.7 macrophages incubated with HEMA. (**B**) Quantitatively, the JC-1 ratio of the ratios of monomers/aggregates is shown. Data are expressed as mean ± SD. The number of parallel measurements is 4. * means that *p* < 0.05 compared with HEMA at the concentration of 0 mM.

Mitochondrial dysfunction leads to apoptosis by disrupting the mitochondrial integrity of cells. According to the results of the JC-1 staining (Figure 5 and Table 2), HEMA-induced mitochondrial dysfunction was concentration-dependent, and significant effects were observed starting from 1 mM (*p* < 0.05).

### 3.6. Effects of HEMA on Intracellular ROS Generation in RAW264.7 Cells

**Figure 6:**
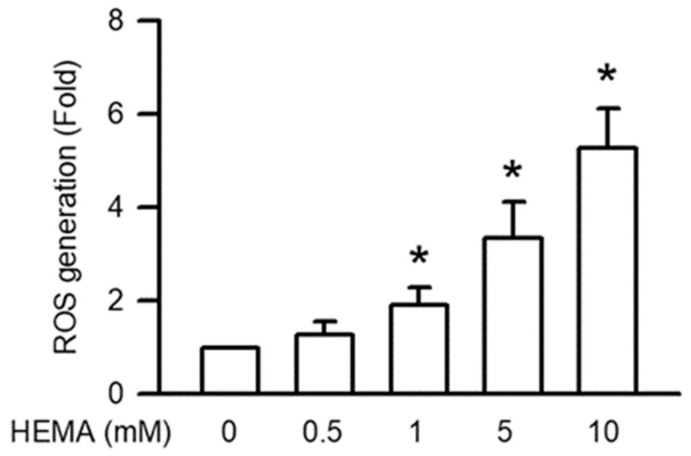
Generation of ROS was induced by HEMA in RAW264.7 cells. After 24 h of treatment with HEMA, DCFH-DA measured ROS generation. Data are expressed as mean ± SD. The number of parallel measurements is 4. * means that *p* < 0.05 compared with HEMA at the concentration of 0 mM.

Intracellular ROS generation plays a major role in apoptosis through mitochondrial dysfunction. As displayed in Figure 6, HEMA induced significant intracellular ROS generation in the RAW264.7 cells in a concentration-dependent manner (starting from 1 mM; *p* < 0.05).

### 3.7. Effects of HEMA on AOE Activity in RAW264.7 Cells

**Figure 7:**
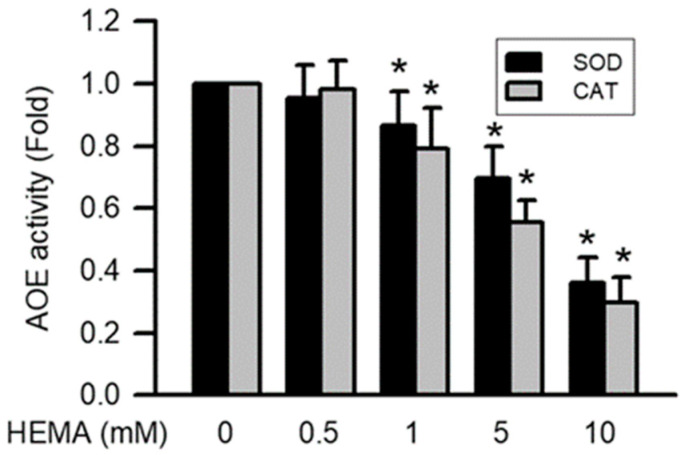
Activation of AOEs was reduced by HEMA in RAW264.7 macrophages. Data are expressed as mean ± SD. The number of parallel measurements is 4. * means that *p* < 0.05 compared with HEMA at the concentration of 0 mM.

The activation of AOEs including SOD and CAT after 24 h of exposure to HEMA at different concentrations was monitored by the SOD and CAT activity assay kit. It was found that after 24 h of exposure to HEMA at different concentrations, the activity of AOEs (including SOD and CAT) significantly decreased depending on the concentration (starting from 1 mM; *p* < 0.05; Figure 7).

## 4. Discussion

HEMA, a water-soluble, low-molecular-weight monomer, enhances the wetting properties and penetration efficacy of demineralized bone matrices of HEMA-based polymers; this property has led to its widespread application in the manufacture of biomaterials for dentistry, orthopedics, and ophthalmology [9,10,22,23]. Previous reports have revealed that HEMA can form polymerized biomaterials in the presence of various solvents and in optimal incubation durations. It can also be eluted from adhesive systems [24,25,26]. Furthermore, HEMA is a derivative of methacrylic acid and acrylic acid, which cause allergic symptoms such as rubefaction, pruritus, persistent paresthesia, and induration [27,28]. HEMA has been reported to induce cytotoxicity in differential macrophages, including alveolar macrophages, J774A.1 macrophages, RAW264.7 macrophages, and human peripheral blood mononuclear cells through apoptosis and not necrosis [15,16,17,18,19,29]. Our findings corroborate those of this previous report; specifically, we observed that HEMA induced cytotoxicity in RAW264.7 macrophages in a concentration-dependent manner through apoptosis and not necrosis.

Apoptosis is usually caused by genotoxicity in macrophages [7,21]. Genotoxicity leads to destructive effects and hampers the integrity of double-stranded or single-stranded DNA [30,31]. The Comet and MN assays are single-cell microgel electrophoresis techniques that help detect single- or double-stranded breaks in the DNA at the individual cell level [30,31]. Genotoxicity was evaluated using HEMA in a concentration-dependent manner in human lymphocytes, gingival fibroblasts, and bronchial epithelial cells [32,33,34]. In addition, HEMA induced DNA fragmentation in RAW264.7 macrophages and human peripheral blood mononuclear cells [29]. In the present study, using the Comet and MN assays, we successfully demonstrated that HEMA induced genotoxicity in macrophages. These results indicate that genotoxicity is the primary process through which HEMA induces cytotoxicity through apoptosis in macrophages.

Caspases are a family of cysteinyl proteases and play a critical role in the regulation of genotoxicity [35]. Caspases are subclassified according to their mechanism of action as follows: extrinsic initiator caspase-8, intrinsic initiator caspase-9, and executor caspase-3 [36]. Extrinsic pathway-initiated apoptosis includes caspase-8 activation mediated by death receptors. Intrinsic pathway-initiated apoptosis includes mitochondrial dysfunction-mediated caspase-9 activation. The activation of caspase-8 and caspase-9 results in the activation of caspase-3 [35,36]. HEMA induces caspase-3 activation in RAW 264.7 macrophages, human gingival fibroblasts, and human dental pulp cells [19,37,38]. HEMA has been reported to induce the activation of caspase-9, and not caspase-8, in rat submandibular salivary gland acinar cells [39]. The results of the present study demonstrate that the activation of caspase-3 and caspase-9 is more sensitive than that of caspase-8 in RAW264.7 cells treated with HEMA. Furthermore, this study revealed that mitochondrial dysfunction, which is the initiation factor of the intrinsic pathway, is induced by HEMA in a concentration-dependent manner in macrophages. On the basis of these findings, we propose that HEMA-induced genotoxicity occurs mainly through the intrinsic caspase pathway, which includes caspase-3 and caspase-9, in macrophages.

Cell cycle progression, cell proliferation, cell differentiation, and other normal physiological functions in mammalian cells are regulated by ROS, including superoxide, nitric oxide, hydrogen peroxide, hydroxyl radicals, and relative free radicals [40,41]. In addition, ROS play a major role in the immune system by defending against pathogens. The overgeneration of ROS is harmful to the peripheral tissues [41,42]. The generation of intracellular ROS is the main reason for mitochondrial dysfunction [43]. Moreover, the accumulation of intracellular ROS is induced by the detoxification system of AOE, which does not maintain low activity [41,43]. The upregulation of ROS generation considerably increases caspase activation and mitochondrial disruption [41]. Previous studies have reported that HEMA induces intracellular ROS generation in macrophages, gingival fibroblasts, and odontoblast-like cells [16,17,18,44]. HEMA also induces the downregulation of SOD expression and the upregulation of CAT expression in macrophages [16,17,18]. We also demonstrated that HEMA induces intracellular ROS generation. Furthermore, the activation of AOEs, including SOD and CAT, is inhibited by HEMA in macrophages. Accordingly, we can infer that HEMA-induced intracellular ROS accumulation results in caspase activation.

## 5. Conclusions

In summary, the present study demonstrated the deleterious effects of HEMA in RAW264.7 macrophages. Exposure to HEMA resulted in decreased cell viability through apoptosis and not necrosis. Moreover, the apoptotic effect of HEMA was found to be associated with genotoxicity. The molecular mechanisms underlying HEMA-induced genotoxicity involve the mitochondrial-dependent intrinsic caspase pathway and intracellular ROS accumulation. Our findings provide a clearer understanding of the mechanisms underlying the cytotoxicity of HEMA via apoptosis and genotoxicity in the macrophages. 

## Figures and Tables

**Table 1:** The parameters of cell responses in RAW264.7 macrophages exposed to the different concentrations of HEMA for 24 h in Annexin V-FITC apoptosis-detection kit.

HEMA (mM)	Viable Cells (%)	Necrotic Cells (%)	Apoptotic Cells (%)
0	97.17 ± 1.02	0.23 ± 0.10	2.83 ± 0.77
0.5	93.70 ± 1.76	0.65 ± 0.19	5.70 ± 0.57
1	91.90 ±2.38 *	0.70 ± 0.28	7.35 ± 2.52 *
5	91.45 ± 1.71 *	0.55 ± 0.24	7.98 ± 1.64 *
10	78.43 ± 4.71 *	1.13 ± 0.75	20.40 ± 4.56 *

* means that *p* < 0.05 compared with HEMA at the concentration of 0 mM.

**Table 2:** The parameters of mitochondrial dysfunction in RAW264.7 macrophages exposed to the different concentrations of HEMA for 24 h via JC-1 staining.

HEMA (mM)	0	0.5	1	5	10
JC-1 monomer (%)	1.53 ± 1.09	3.53 ± 1.31	4.83 ± 0.85 *	6.03 ± 1.23 *	9.68 ± 2.29 *
JC-1 aggregates (%)	98.20 ± 1.24	96.20 ± 1.64	93.83 ± 0.88 *	92.73 ± 0.94 *	89.33 ± 1.25 *
JC-1 ratio	0.02 ± 0.01	0.04 ± 0.01	0.05 ± 0.01 *	0.06 ± 0.01 *	0.11 ± 0.03 *

* means that *p* < 0.05 compared with HEMA at the concentration of 0 mM.

## Data Availability

The data presented in this study are available on reasonable request from the corresponding author.

## Abbreviations

AOE	antioxidative enzyme
CAT	Catalase
Comet	alkaline single-cell gel electrophoresis
DCFH-DA	dichlorofluorescin diacetate
DMEM	Dulbecco’s modified Eagle’s medium
DMSO	dimethyl sulfoxide
FBS	Fetal bovine serum
HEMA	2-Hydroxyethyl methacrylate
MN	Micronucleus
MTT	3-(4,5-dimethylthiazol-2-yl)-2,5-diphenyl tetrazolium bromide
ROS	reactive oxygen species
